# Effectiveness of oral health education intervention among 12–15-year-old school children in Dharan, Nepal: a randomized controlled trial

**DOI:** 10.1186/s12903-021-01877-6

**Published:** 2021-10-14

**Authors:** Krishna Subedi, Ashish Shrestha, Tarakant Bhagat, Dharanidhar Baral

**Affiliations:** 1 Dental Department, Pokhara Academy of Health Sciences, Pokhara, Nepal; 2 Department of Public Health Dentistry, College of Dental Surgery, B.P. Koirala Institute of Health Sciences, Dharan, Nepal; 3 School of Public Health and Community Medicine, B.P. Koirala Institute of Health Sciences, Dharan, Nepal

**Keywords:** Effectiveness, Oral health education, School children

## Abstract

**Background:**

School-aged adolescents are in particular need of preventive program to ensure positive long-term oral health and hygiene. The objective of this study was to assess the effectiveness of an oral health education (OHE) intervention on oral hygiene knowledge, attitude and practices (KAP), plaque control and gingival health among 12–15 years old school children in Dharan sub-metropolitan city, Nepal.

**Methods:**

A randomized controlled trial was conducted with parallel study groups, comprising 12–15-year-old school children, 120 in each group. OHE was given to the experimental group at baseline, third and sixth months and to the control group after completion of the study. Interview of the participants were done using a 23-item questionnaire for assessment of oral hygiene KAP. For each question, correct answer was scored as 1 and wrong answer was scored zero. An overall composite score was then created, by adding the individual scores. Oral examination was done using mouth mirror and WHO probe to record Turesky–Gilmore–Glickman modification of the Quigley-Hein plaque index, Gingival index and Dentition status and treatment needs. Analysis was done using chi-square test for categorical data and independent t test, Mann–Whitney U test, repeated measures ANOVA and post hoc Tukey’s test for quantitative data. The level of significance was set at *P* < 0.05.

**Results:**

There was 54.58% improvement in overall oral hygiene KAP in experimental group (*P* = 0.001) whereas no improvement was seen in control group at the end of the study. The mean plaque score was improved by 57.67% (*P* = 0.001) in experimental group in comparison to 4.56% in control group. Gingival index was improved by 49.90% (*P* = 0.001) in experimental group in comparison to 0.7% in control group. Caries experience was increased in both groups but no significant difference was seen.

**Conclusions:**

The study concluded that oral health education was effective in improving oral hygiene KAP, plaque control and gingival health.

*Trial registration* The trial was retrospectively registered with Clinical Trial Registry India (CTRI) with identifier no. CTRI/2018/05/013985, registered on 05/21/2018. (http://www.ctri.nic.in/Clinicaltrials/pdf_generate.php?trialid=23651&EncHid=&modid=&compid=%27,%2723651det%27). Institutional Review Committee, B. P. Koirala Institute of Health Sciences (BPKIHS), Dharan, Nepal provided the ethical approval (Ref. No.: 292/074/075-IRC).

## Background

The worldwide prevalence of dental disease is a constant reminder of the universal need for effective dental health education programs [1]. World Health Organization (WHO) reports 70–95% of school-aged children have experienced dental caries in South-East Asia [2]. Health education is a key strategy in the process of acquisition of behaviors that promote and maintain health [3]. Mastrantonio and Garcia point out that it is possible to transform negative attitudes into healthy habits to the population through education [3]. Oral health is indispensable to general health and quality of life [4, 5]. Oral health is multifaceted and includes the ability to speak, smile, smell, taste, touch, chew, swallow, and convey a range of emotions through facial expressions with confidence and without pain, discomfort, and disease of the craniofacial complex [6].

Few aspects of health which are accessible to personal control such as oral hygiene, can be improved by simple behavioral changes. The objective of Oral health education (OHE) program is to improve the knowledge and oral hygiene status of the participants which would have obvious merits. As a result of OHE, there has been improvement of self-reported oral health related practices and behavior as well as clinical parameters of oral health such as oral hygiene, gingival health and dental caries [7].

Many of the oral diseases in the advanced stages lead to pain, discomfort and handicap. In a cross-sectional survey of 106 schools in different parts of Nepal, 45% of 4770 school children from age 8 to 14 years experienced toothache. Major impact included inability to eat (61%) loss of sleep (14%), financial burden (7%), inability to play (6%), missed school (5%), inability to do homework (2%) and all the above (6%) [8].

About 37 percent of the total population of Nepal is in between 5 to 14 years, and out of them nearly 92% children are enrolled in school [9].The 2004 National Pathfinder Survey of Nepal showed that 41% of 12–13 years old schoolchildren suffer from dental caries with mean DMFT of 1.1 [8]. Prevalence of dental caries among public school children in eastern Nepal is 60.30% in primary dentition and 55.6% in permanent dentition [10]. A study in eastern Nepal found fair plaque control in 62.3% and poor plaque control in 37.7% in 10–13 years school children. It concluded that a community based oral health intervention program was necessary [11].

Oral health education and promotion can be delivered at different forums like hospitals, primary health care centers, private dental clinics, school, etc. [7]. However, schools are perhaps the best place for promoting oral health because schools form an ideal setting by offering an efficient and effective way to reach over 1 billion children worldwide and through them, their families as well as communities [4, 5, 7]. School based approach seems to be more productive in delivering preventive and curative services as compared to community-based approach. Children who are suffering from poor oral health are 12 times most likely to have restricted daily activities including missing schools as compared to those who have good oral health [4, 5, 7]. Due to this more than 50 million school hours are lost annually which could lead to negative impact on the long-term performances of children at school and success in future [4, 5, 7].

In recent years, attention has been drawn toward assessing the effectiveness of oral health education programs [12]. This is in line with demand for evidence-based research and will help to inform policy makers on how to allocate resources [12]. A number of systematic reviews have been conducted on the available evidence [12, 13]. These have shown that oral health education can be effective in increasing knowledge in the short term and to some extent, behavior such as tooth brushing and healthy eating [12–14]**.**

To the best of our knowledge no such educational intervention studies have been published till now in Nepal. In a country like Nepal where majority of people who don’t have awareness and accessibility to oral-dental care, there is strong and urgent need for oral health education intervention which will help to reduce mortality and morbidity. The objective of this study was to assess the effectiveness of an oral health education intervention on oral hygiene knowledge, attitude and practices (KAP), plaque control and gingival health among 12–15 years old school children in Dharan sub-metropolitan city, Nepal.

## Methods

### Trial design and study participants

This was a randomized controlled concurrent parallel trial conducted from October 2017 to September 2018. In concurrent parallel trial comparisons are made between two randomly assigned groups with one group exposed to intervention. Children belonging to 12–15-year-old age group studying in grade 8 and 9 in public and private schools of Dharan sub-metropolitan city, Nepal were included in the study.

Dharan is a sub-metropolitan city in Sunsari district of province No.1, Nepal. The total area of the sub-metropolitan is 192.32 square kilometers. According to 2011 Census conducted by Central Bureau of Statistics (CBS), Dharan Sub-Metropolitan City had total population of 137,705 with 64,671 males and 73,034 females. There were only 106,424 people fully literate as of 2011 who were able to both read and write, while 2349 people were able to read but not write. According to Nepal Government records as of 2017, there were total 6,515 school children studying in grade 1 to 12 in Dharan Sub-Metropolitan City with 3128 (48.01%) of males and 3387 (51.99%) females. The shortest distance from capital city (Kathmandu) of Nepal to Dharan through road is 379 km which takes around 8 h and 35 min.

### Ethical considerations and trial registration

Ethical approval for the study was obtained from the Institutional Review Committee, B.P.Koirala Institute of Health Sciences (BPKIHS), Dharan (Ref. No.: 292/074/075-IRC and Code No: IRC/1086/017). Approval was also obtained from Thesis Protocol Evaluation Committee of BPKIHS, Dharan (Ref. No.: Acd/978/074/075). The study was registered as a clinical trial (www.ctri.nic.in) in the Indian Council of Medical Research (ICMR)- National Institute of medical Statistics; the Clinical Trial Registry India identifier no. CTRI/2018/05/013985 (http://ctri.nic.in/Clinicaltrials/rmaindet.php?trialid=23651&EncHid=57035.73346&modid=1&compid=19). It was retrospectively registered on 05/21/2018. Official permission was obtained from the Dharan sub-metropolitan city and the concerned school authorities before commencing the study. A written informed consent was obtained from all parents of the study participants and verbal assent from each child.

### Eligibility criteria

Secondary schools providing co-education were included in this study. Co-education means school providing education to both boys and girls. Cooperative 12–15 years old school children studying in grade 8 and 9 whose parents gave their written informed consent were included in this study. Cooperative means those children who had provided the verbal assent and given permission for oral examination.

Children with any systemic disease, requiring any emergency dental treatment and with orthodontic appliances were excluded.

### Randomization

Total 18 public schools and 42 private schools of Dharan sub-metropolitan city met the inclusion criteria. After getting verbal permission from the principals of the schools, 4 public and 8 private schools (20% of total) were randomly selected using lottery method, by an assistant who was not participating in the field study. A randomization master list was prepared based on computer generated random numbers and each school was assigned to a group (Group 1, Group 2) by a biostatistician. Allocation concealment were done using opaque envelope methods.

Systematic random sampling was done to include the students from the schools in each study group. Number of students from type of schools (public or private) and each school was selected on the basis of population proportion ratio.

### Blinding

Coding was given as 1 and 2 to the 2 different groups. It was not revealed during the data entry time and analysis time. The two groups 1 and 2 were revealed as control and experimental respectively only after completing the analysis.

### Sample size

This study considered (95% CI) and 80% power to estimate the sample size. For this purpose, mean ± SD (gingival index) value of intervention group (2A) 0.78 ± 0.42 and mean ± SD value of control group (1B) 0.94 ± 0.3822 respectively were taken. Therefore, mean of control group (µ1) = 0.94, mean of intervention group (µ2) = 0.78 and average standard deviation of control and intervention group (ϭ) = 0.40 [15].Using following formula, Sample Size (n) = (2 ϭ^2^ (z_α/2_ + z_β/2_)^2^)/(µ1 − µ2)^2^, the sample size was calculated as 98. Considering 20% attrition rate total sample size was increased to 120 in each group.

### Questionnaire

The questionnaire contained pretested standardized closed ended questions which were selected from previous researches [16–18] (Annexure 1). Face and content validity of the structured questionnaire was done by three subject experts. A 23-item questionnaire was translated and validated in Nepali language (local language) through standard back translation method. Test–retest was used to check the reliability and internal consistency of the questionnaire. Cronbach’s alpha value of 0.81 showed good internal consistency of the questionnaire.

Each of the 23 multiple choice questions had a single correct answer. All questions had a binary outcome which was coded as one for correct and zero for incorrect. Every correct answer in baseline, 3 and 6 months after intervention was scored as 1 and wrong answers were scored zero. An overall composite score was then created, by adding the individual scores on each question. The highest possible score for oral health knowledge was 11 for each individual. The highest possible score for oral health practices and attitude were 8 and 4 respectively. The highest possible overall score for oral hygiene KAP was 23. The mean score was then calculated for each group and then compared. The percentage change was calculated by subtracting the pre-test percentage from the post-test percentage [100 × (baseline mean score-6 months score)/baseline score].

Face to face interview of the participants were done by the single investigator (KS). Time taken for each interview was 4–5 min. Demographic variables included age, sex, grade, type of school (public or private) and socioeconomic status (SES). SES was calculated using Kuppuswamy scale and classified as per the modifications done in the year 2009 [19] using current consumer price index for the year 2017. The current consumer price index was obtained online from Nepal Rastra Bank website (Nepal RB 2017) and the conversion factor was calculated (Conversion factor = consumer price index 2017 divided by consumer price index of 1976) [20]. The computed conversion factor was 26.7 (114.8/4.3). For simplicity, SES was categorized into upper (26–29), middle (11–25) and lower (≤ 10) class.

### Clinical examination

Clinical oral examination was done according to WHO basic oral health surveys methods [21]. A pilot study was conducted among 25 participants of similar school children with similar age, grade and socioeconomic status, who were not involved in the main study, for training and calibration of the examiner, feasibility assessment of the study and the reliability of questions.

All children were examined at their schools, lying on a bench with the examiner seated behind the subject’s head, under artificial light. Oral examination was carried out by using sterilized instruments including mouth mirror, WHO probe and disposable gloves. Oral examinations were done to record Turesky–Gilmore–Glickman modification of the Quigley-Hein plaque index [22], Gingival index [23] and Dentition status and treatment needs at baseline and 3rd and 6th months of the study period. DMFT were calculated from dentition status and treatment needs.

Duplicate examinations were performed among 25 participants during the study to test the intra-examiner reliability which was measured by interclass correlation coefficient (ICC).

### Intervention

Oral health education included topics like importance of teeth, type of dentition, brushing and flossing techniques and dental caries—its etiology, signs and symptoms, complications, preventive methods, the role of fluorides, plaque and calculus and its effect on gingival and periodontal health, diet and nutrition, importance of oral health to general health. OHE was first provided in one school which was not considered in the main study for the validation of the OHE materials. OHE was provided by KS and supervised by AS and TKB**.**OHE using tooth models and PowerPoint presentation was given to 12–15 children in a single session of 30 min in each follow up to the experimental group by KS.

No OHE was given to the control group. In the experimental group, reinforcement of OHE was done at the 3rd and 6th months. To avoid contamination, only one group (either control or experimental) was included from one school. After completion of study (after 6 months) the same OHE that was given to the experimental group was given to the control group. During first and second follow up time maximum 3 visits to every schools was done to include the maximum number of children. Those children who were not present during examination at follow up periods were considered as missing.

### Evaluation of intervention

Baseline assessment was done in January–February 2018, second and third assessment was done in April–May and August 2018 respectively. On each visit oral health education was given as intervention for experimental group.

Intervention was evaluated by assessing the improvements in oral hygiene knowledge, practice and attitude (correct answers) and changes in plaque and gingival scores in experimental group compared with the control group.

### Outcomes

The primary outcome measures were change in mean score of oral hygiene KAP, plaque control, gingival health and DMFT after intervention at 3 and 6 months in experimental and control group.

### Statistical analysis

After completion of the trial, data obtained were entered in Microsoft Excel Sheet version 2007 and analyzed using the Statistical Package for Social Sciences (SPSS version 11.5). The level of significance was set at *p* ˂ 0.05. Intra-examiner reproducibility for coding was measured by intra-class correlation coefficient (ICC).

Descriptive analysis was performed to summarize the clinical and socio-demographic characteristics of each group at baseline in order to assess how comparable the groups were at beginning of the study. Descriptive statistics including the mean, median and standard deviations were computed for oral hygiene KAP, plaque index (PI), gingival index (GI) and DMFT.

Chi-square test was used to find the significance of study characteristics on categorical scale. Repeated-measures ANOVA was used to find the significance of oral hygiene knowledge, practice and attitude, plaque index and gingival index scores between two groups at baseline, 3 and 6 months respectively. For significant repeated measures ANOVA post hoc Tukey’s test was used. Independent t test was used to find the pairwise significance between the groups regarding oral hygiene KAP, plaque index and gingival index scores. Mann–Whitney u test was used to find the pairwise significance between 2 groups regarding mean DMFT.

## Results

During the study intra-examiner reproducibility was assessed among 25 randomly selected participants by doing duplicate examination. While using intra-class correlation coefficient intra-examiner reliability for GI was 0.98 and 0.91 for PI. The Cronbach alpha value for the oral health knowledge, attitude and practice was found to be 0.89 (good reliability), 0.93 (excellent reliability) and 0.93 (excellent reliability) respectively.

**Fig. 1:**
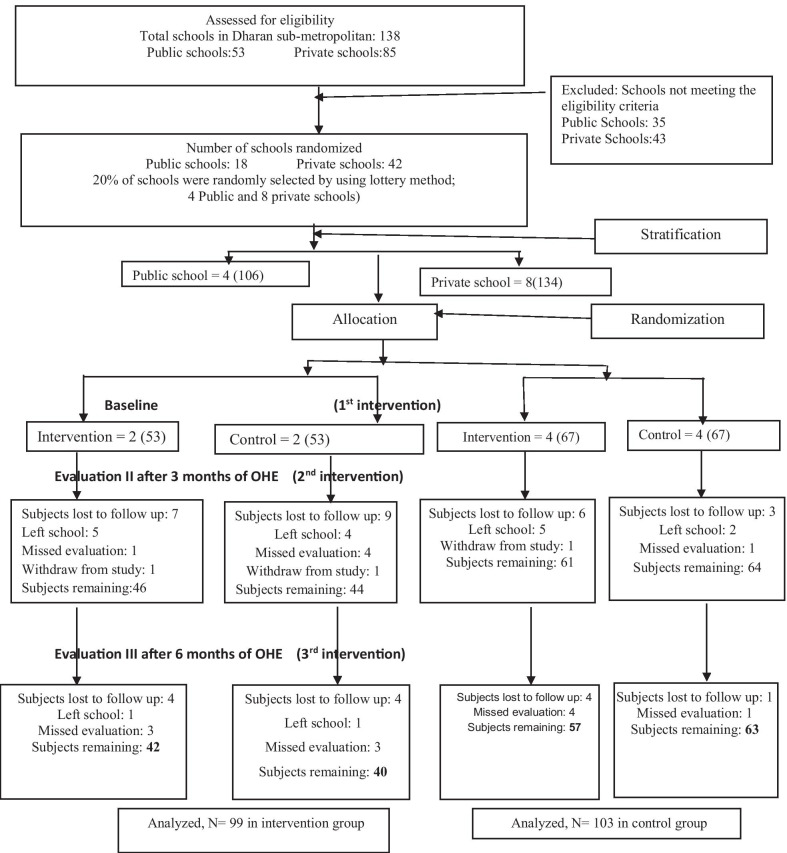
CONSORT flowchart of the children screened for the study

There were total of 240 school children (120 male and 120 female) allocated into two groups with a mean age of 14.25 ± 0.73 years. Figure 1 presents the CONSORT flow diagram tracking subject participation for the entire study. The dropout rate was 17.5% for experimental and 14.16% for control groups. Ninety-nine from experimental and 103 from control groups completed the study.

Overall, 49.5% male and 50.5% female completed the study with mean age of 14.39 ± 0.65 and 14.11 ± 0.79 in experimental and control group respectively. Gender (*P* = 0.78), grade (0.47) and type of school (*P* = 0.60) did not show any significant differences in both the groups. More number of school children belonged to medium socioeconomic status (*P* = 0.009) and 14–15 age groups (*P* = 0.02) in experimental group as compared to control group (Table 1).

**Table 1:** Distribution of a sample of 12–15-year-old students in Nepal (N = 202)

Age in years	Experimental group N = 99 (%)	Control group N = 103 (%)	Pearson chi-square	*P* value
Mean age	14.39 ± 0.65	14.11 ± 0.79		
12–13	9 (9.1%)	21 (20.4%)	5.09	**0.02**
14–15	90 (90.9%)	82 (79.6%)		
Sex				
Male	50 (50.5%)	50 (48.5%)	0.07	0.78
Female	49 (49.5%)	53 (51.5%)		
Grade				
8	54 (54.5%)	51 (47.6%)	0.51	0.47
9	45 (45.5%)	52 (52.4%)		
Socioeconomic status				
Low	32 (32.3%)	52 (50.5%)	6.85	**0.009**
Medium	67 (67.7%)	51 (48.5%)		
Type of School				
Public	42 (42.4%)	40 (38.8%)	0.27	0.60
Private	57 (57.6%)	63 (61.2%)		

*P* value in bold letter is statistically significant

Table 2 shows the intragroup and intergroup comparison of oral hygiene knowledge, practices, attitude and overall oral hygiene KAP in 2 groups (experimental and control group). There were no significant differences between 2 groups regarding oral hygiene knowledge, practices, attitude and overall oral hygiene KAP at the baseline. However, the intergroup comparison showed the significant improvements in the experimental group at 3 and 6 months except the oral hygiene attitude at 3 months. Oral hygiene attitude was improved at 3 months in experimental group but not statistically significant (*P* = 0.130). But it was found to be statistically significant at 6 months (*P* = 0.001). Regarding oral hygiene knowledge, intragroup comparison showed that there was 77.51% improvement in experimental group (*P* = 0.001**)** and 6.6% improvement in the control group (*P* = 0.107) at the end of 6 months. Regarding intragroup comparison of oral hygiene practice, 31.87% improvement was seen in experimental group and 7.82% reduction in control group. Oral hygiene attitude improved by 35.93% in experimental group and decreased by 4.94% in control group at 6 months. The overall oral hygiene KAP improved by 54.58% in experimental group whereas it was found almost similar to the baseline in control group at 6 months.

**Table 2:** Intragroup and intergroup comparisons of knowledge, attitude, and practice (KAP) regarding on oral hygiene in control and experimental group at baseline, 3 and 6 months after intervention (N = 202)

	Experimental group (mean ± SD)	Control group (mean ± SD)	T value	*P*^#^ value
Mean oral hygiene knowledge				
Baseline	5.47 ± 1.80	5.75 ± 2.0	1.015	0.312
3 months	8.47 ± 1.52	6.07 ± 1.78	− 10.263	0.001
6 months	9.71 ± 1.10	6.13 ± 1.71	− 17.748	0.001
F value	253.689	2.302		
*P*^∆^ value	0.001	0.107		
% change	77.51	6.6		
Mean oral hygiene practice				
Baseline	3.42 ± 1.34	3.45 ± 1.144	0.127	0.899
3 months	4.34 ± 1.45	3.46 ± 1.15	− 4.799	0.001
6 months	4.51 ± 1.03	3.18 ± 1.31	− 7.926	0.001
F value	32.215	2.254		
*P*^∆^ value	0.001	0.108		
% change	31.87	− 7.82		
Mean oral hygiene attitude				
Baseline	2.56 ± 0.84	2.63 ± 0.86	0.627	0.531
3 months	2.89 ± 0.79	2.72 ± 0.79	− 1.522	0.130
6 months	3.48 ± 0.69	2.50 ± 0.79	− 9.368	0.001
F value	47.375	3.291		
*P*^∆^ value	0.001	0.039		
% change	35.93	− 4.94		
Overall oral hygiene KAP				
Baseline	11.45 ± 2.52	11.83 ± 2.80	0.325	0.371
3 months	15.72 ± 2.48	12.24 ± 2.55	− 9.790	0.001
6 months	17.70 ± 2.03	11.82 ± 2.70	− 17.520	0.001
F value	286.823	1.472		
*P*^∆^ value	0.001	0.232		
% change	54.58	− 0.084		

^#^Independent t test

^∆^Repeated ANOVA

Table 3 shows that there was significant improvement in oral hygiene knowledge, attitude and overall oral hygiene KAP at 3 and 6 months. Regarding oral hygiene practice significant improvement was seen from baseline to 3 and 6 months. However no significant changes were seen in between 3 and 6 months.

**Table 3:** Comparison of mean knowledge, attitude, and practice (KAP) at different time intervals using post hoc Tukey’s test among a sample of 12–15-year-old students in Nepal (N = 202)

	Time intervals		Mean difference ± SE	*P* value	95% confidence interval	
					Lower bound	Upper bound
Oral hygiene knowledge	Baseline	3 months	− 3.00 ± 0.20	< 0.001	− 3.41	− 2.58
		6 months	− 4.23 ± 0.19	< 0.001	− 4.61	− 3.84
	3 months	6 months	− 1.23 ± 0.17	< 0.001	− 1.58	− 0.88
Oral hygiene practice	Baseline	3 months	− 0.91 ± 0.14	< 0.001	− 1.19	− 0.64
		6 months	− 1.08 ± 0.15	< 0.001	− 1.37	− 0.78
	3 months	6 months	− 0.16 ± 0.14	0.27	− 0.45	0.12
Oral hygiene attitude	Baseline	3 months	− 0.33 ± 0.09	0.001	− 0.52	− 0.14
		6 months	− 0.92 ± 0.10	< 0.001	− 1.13	− 0.72
	3 months	6 months	− 0.59 ± 0.09	< 0.001	− 0.77	− 0.41
Overall KAP	Baseline	3 months	− 4.26 ± 0.26	< 0.001	− 4.78	− 3.74
		6 months	− 6.24 ± 0.27	< 0.001	− 6.78	− 5.69
	3 months	6 months	− 1.98 ± 0.26	< 0.001	− 2.49	− 1.46

Table 4 shows that there were no significant differences in mean plaque scores at baseline between experimental and control group. Intergroup comparison showed that plaque score significantly improved in experimental group at 3 and 6 months compared to control group. Intragroup comparison showed that plaque scores was significantly improved in both group at 6 months. Plaque score was improved by 57.67% in experimental group and 4.56% in control group at 6 months.

**Table 4:** Intragroup and intergroup comparisons of mean plaque index and gingival index scores in control and experimental group (N = 202)

	Experimental group (mean ± SD)	Control group (mean ± SD)	T value	*P*^#^ value
Mean plaque index scores				
Baseline	2.15 ± 0.52	2.19 ± 0.41	0.493	0.622
3 months	1.44 ± 0.46	2.23 ± 0.49	11.687	0.001
6 months	0.91 ± 0.40	2.09 ± 0.53	17.558	0.001
F value	275.518	3.732		
*P*^∆^ value	0.001	0.026		
% change	57.67	4.56		
Mean gingival index scores				
Baseline	1.32 ± 0.25	1.36 ± 0.21	1.192	0.235
3 months	0.86 ± 0.30	1.30 ± 0.26	11.123	0.001
6 months	0.78 ± 0.31	1.35 ± 0.22	14.793	0.001
F value	190.801	2.948		
*P*^∆^ value	0.001	0.05		
% change	40.90	0.7		

^#^Independent t test

^∆^Repeated ANOVA

Table 4 shows that there were no significant differences in GI at the baseline between experimental and control group. Intergroup comparison showed that GI was significantly improved at 3 and 6 months in experimental group. Intragroup comparison showed that significant improvement in mean GI scores at 6 months in experimental group. There was 40.90% improvement in mean GI in the experimental group and only 0.7% improvement in the control group.

Table 5 shows that plaque control and mean GI scores was significantly improved at 3rd and 6th months in experimental group. However, in control group significant plaque control was seen only from 3rd to 6th months.

**Table 5:** Comparison of mean plaque scores and gingival scores at different time intervals using post hoc Tukey’s test among a sample of 12–15-year-old students in Nepal (N = 202)

	Time intervals		Mean difference ± SE	*P* value	95% confidence interval	
					Lower bound	Upper bound
Plaque scores in experimental group	Baseline	3 months	0.71 ± 0.05	< 0.001	0.60	0.82
		6 months	1.24 ± 0.05	< 0.001	1.13	1.34
	3 months	6 months	0.52 ± 0.05	< 0.001	0.42	0.63
Plaque scores in Control group	Baseline	3 months	− 0.04 ± 0.05	0.36	− 0.15	0.05
		6 months	0.101 ± 0.05	0.06	− 0.006	0.20
	3 months	6 months	0.073 ± 0.03	0.02	0.007	0.13
Gingival scores in experimental group	Baseline	3 months	0.46 ± 0.02	< 0.001	0.40	0.52
		6 months	0.53 ± 0.02	< 0.001	0.48	0.59
	3 months	6 months	0.07 ± 0.03	0.02	0.007	0.13

Table 6 shows that there was no significant difference in mean and median DMFT between experimental and control group at the baseline, 3 and 6 months.

**Table 6:** Mean, median and IQR of DMFT at baseline, 3 months and 6 months after intervention in control and experimental group (N = 202)

Group	Baseline			3 months			6 months			% change
	Mean ± SD	Median	IQR	Mean ± SD	Median	IQR	Mean ± SD	Median	IQR	
Experimental group	0.78 ± 1.41	0	1	1.41 ± 2.45	1	2	1.55 ± 2.87	1	2	98.71
Control (N = 103)	0.92 ± 1.52	0	1	1.80 ± 3.55	1	2	2.23 ± 4.27	1	3	142.39
U value	4792.00			4906.00			4633.50			
P^γ^	0.413			0.623			0.237			

^γ^Mann–Whitney U test

## Discussion

The results of the present study showed that OHE seems to be effective in increasing oral hygiene KAP and improving plaque control and gingival health.

In the present study 12–15-year-old school children were taken as study population because it is likely that by this age all the permanent teeth except the third molars will have erupted. Therefore, age 12 years has been a global indicator for comparisons and surveillance of disease trends at international level [17, 24]. Proper use of a toothbrush requires a certain degree of dexterity and skill. Nonetheless, children as young as 11 years of age have the ability to brush effectively [25]. It was expected that at this age student can clearly understand the subject being taught to them. They have enough manual dexterity to master the proper technique of brushing [26]. They are in the very influential stages of life; the habits, beliefs, skills and attitudes that have developed would tend to last longer [27]. A study conducted by Ingle et al. found that eight years old children are not appropriate group to start with oral health education intervention as they could not follow the oral hygiene instructions properly [28].

The results showed that oral hygiene knowledge was significantly increased by 77.51% in experimental group and 6.6% in control group when compared to baseline. It was almost similar to the study done by Rajesh et al. [29] where oral hygiene knowledge was increased by 57.25% versus 0.80% in computer method of oral education vs control group at 3 months. In Walsh study [30] dental health knowledge was increased by 44.80% versus 5.49% in experimental and control group. A study conducted by Haque et al. [7] found that oral health knowledge was significantly increased from 19.3% (baseline) to 75.9% at 6 months after intervention. Similarly, the oral health knowledge was increased after intervention in experimental group in the studies conducted by Al Saffan et al. [31] and D'Cruz et al. [32]. Oral hygiene attitude and practices was significantly improved by 35.5% and 31.87% respectively in experimental group whereas it was decreased by 4.94% and 7.82% in control group. The findings of this study were in accordance with the study conducted by Haque et al. [7] where oral health attitude and practices was significantly increased by 43.1% and 36.2% respectively in the intervention group after 6 months. Similarly, the oral health attitude and practices were significantly increased in the study conducted by Sanadhya et al. [18] after intervention. The overall oral hygiene knowledge, attitude and practices was significantly increased by 54.58% in experimental group and almost no change in control group.

There was significant reduction in mean plaque index scores (57.67%) in experimental group whereas only 4.56% reduction in control group. These findings were found to be in accordance with the studies done by Shahapur et al. [26], Lakshmi et al. [33], Worthington et al. [34], Gauba et al. [35], Redmond et al. [36], Sharma et al. [37] and Ajithkrishnan et al. [38] where significant reduction in mean plaque levels was reported after intervention. In contrast to this study Frencken et al. [39] and Palenstein et al. [40] found no significant reduction in plaque scores after intervention.

Ganesh et al. [41] reported significant reduction of 17.5% mean plaque level and 27.8% mean GI after 4 weeks of intervention. In a study by Bhardwaj et al. [42] mean plaque and gingival score decreased significantly after intervention irrespective of gender in 12- and 15-years old school children. Damle et al. [43] reported that mean PI and GI was significantly improved after 3 months intervention in 12–15-year-old school children.

There was significant reduction in mean gingival index (40.90%) in experimental group whereas almost no changes (0.7% reduction) in control group after 6 months of oral health education. These findings were found to be in accordance with the studies done by De Farias et al. [44], Gauba et al. [35] and Sharma et al. [37] where significant reduction in mean GI score was reported after intervention. In contrast to this study Ajithkrishnan et al. [38] found no significant reduction in gingival scores after intervention.

The present study did not show any significant changes in mean DMFT in between and among the groups. The percentage increase in mean DMFT was higher in control group (142.39%) as compared to experimental group (98.71%). It was similar to study conducted by Vanobbergen et al. [45] where mean DMFT was found to be higher in the control group but not significantly different. A study conducted by Frencken et al. [39] reported that mean caries increment in experimental (0.04) and control group (0.19) was not found to be statistically significant over a period of 3.5 years. Sharma et al. [37] and Palenstein et al. [40] also reported similar findings. In contrast to this study Hausen et al. [46] found mean DMFS increments were significantly lower for experimental group than control group after average follow up of 3.4 years. Reason for this difference may be due to longer duration of their study and provision of patient-centered regimen for caries control.

Improvement in oral hygiene knowledge and plaque score was seen in control group. This may have occurred as a result of Hawthorne effect [32, 44, 47]. Hawthorne effect is a form of reactivity whereby subjects improve an aspect of their behavior being studied, and not in response to any particular experimental manipulation [47]. These changes may have occurred because of interest of some children to gain knowledge about various aspects of oral health through varied sources [44, 47]. The mere presence of dentist in the school and possibility of greater attention provided to the students and a questionnaire is likely to have some influence in motivation for better self-care [32, 44].

### Strengths and limitations

Random sampling technique for selection of school and children is the strength of the study which accounts for representativeness and generalizability of the study among school children of Dharan, Nepal.

Since examiner was not blinded, observational bias might have occurred. No environmental factors and lifestyle changes were taken into consideration. Inherent bias i.e. over-reporting of favorable behaviors (related to oral hygiene practices) can be expected. Difference on the age and the SES at the baseline may have compromised the results. Short follow up is also the limitation of the study.

## Conclusions

This study concluded that school based oral health education is effective in improving oral hygiene knowledge, attitude and practices that leads to better plaque control and better improvement in the gingival health. Repetition and reinforcement of oral health education program plays a key role in sustainability of oral health behavior.

### Recommendations

The study showed the effectiveness of oral health education program in improving oral hygiene knowledge, attitude and practices, plaque control level and better gingival health in school children of Dharan, Nepal. A further large-scale trial with longer duration of the study should be conducted throughout the country to confirm the findings of this study. School based oral health education program is easy to organize and inexpensive which can improve the oral hygiene cleanliness and gingival health among school children. Such oral health education intervention programs should be included in the academic curriculum of the school which would be effective in developing country like Nepal.

## Data Availability

The datasets analyzed during the current study are available from the corresponding author on reasonable request.

## Abbreviations

ANVOA: Analysis of variance
BPKIHS: B. P. Koirala Institute of Health Sciences
CI: Confidence Interval
CTRI: Clinical Trial Registry India
DMFT: Decayed Missing Filled Tooth
DMFS: Decayed Missing Filled Surface
GI: Gingival Index
ICC: Intraclass correlation coefficient
KAP: Knowledge Attitude and Practice
OHE: Oral Health Education
PI: Plaque Index
SD: Standard Deviation
SES: Socioeconomic Status
SPSS: Statistical Package for Social Sciences
WHO: World Health Organization
